# The societal impact of electronic sport: a scoping review

**DOI:** 10.1007/s12662-021-00784-w

**Published:** 2021-11-26

**Authors:** Paolo Riatti, Ansgar Thiel

**Affiliations:** Institute of Sports Science, Eberhard Karls University Tübingen, Wilhelmstraße 124, 72074 Tübingen, Germany

**Keywords:** Gaming, Socializing, Addiction, Electronic sport, Societal impact, Scoping review

## Abstract

Competitive computer gaming, known as electronic sport or esport, is growing and professionalizing profoundly during the past years with experts struggling to allocate it in society. This scoping review explores existing evidence and identifies potential societal impact of esport by applying the Mapping Elite Sports Societal Impact Model. Main findings included insights on the motivation of passive and active esport consumption, beneficial socializing, pedagogical or educational aspects, hegemonial clinical pictures in esport, differing popularity regarding demographics and games, and potential interference of the esport economy in traditional sports. The findings implicate a paradigm shift in the world of sport. It can be stated that esport affects society in a positive and a negative way, although the fragmented body of research has only given superficial evidence so far. Future research needs to go into detail regarding the peculiarities and find approaches of isolating the positive aspects, while reducing the negative spin-offs and allow a well-regulated handling of esport on a broad societal level.

**Supplementary Information:**

The online version of this article (10.1007/s12662-021-00784-w) contains supplementary material, which is available to authorized users.

## Introduction

In modern society sport has become an integral part of everyday life. This rooting ranges far beyond participation as leisure or health care activities, but has differentiated into a vital economic sector, a philosophy of life and affects everyday interaction such as behavior or speech (Stichweh, 2013). It is usually positively connoted and is regarded as an engine for development, thus underlining that sport has an impact on society on many levels (De Bosscher, Shibli, & De Rycke, 2021; Pawlowski, Schüttoff, Downward, & Lechner, 2018; Spaaij, 2009). The depiction of sport has evolved throughout history and differs from culture to culture. It evolves and adapts to trends and changes in society (Heinemann, 2007). Nowadays, digitalization is a major driver of change in society and therefore also in sport (Miah, 2014; Ratten, 2019). As the digital development in sport grows, it also alters the social depiction and role of sport (Thiel & Gropper, 2017). Therefore, sport undergoes a variety of changes, like improved equipment such as the video assistant referee in football, big data usage for health and performance diagnostics, or an alteration of movement practices (Edgar, 2019; Thiel, Seiberth, & Mayer, 2013). Several reviews have shown how digitalization changes common practices in sport (Abeza, O’Reilly, Séguin, & Nzindukiyimana, 2015; Baca, Dabnichki, Heller, & Kornfeind, 2009; Filo, Lock, & Karg, 2015; Gruettner, 2019; Rigamonti et al., 2020; Xiao et al., 2017). But there is still little knowledge about electronic sport (esport), a symbiosis of gaming and sportive competition, which has seen a considerably strong growth since the 1990s, especially due to an evolving digitalization and a growing computer game industry. It is a global phenomenon, particularly popular in the far east, like China or South Korea, Europe, North America or Brazil (Parshakov & Zavertiaeva, 2018; Taylor, 2012). Regarding player base, spectatorship, or prize money, it has outperformed many traditional sports and witnessed an additional boom during the coronavirus disease 2019 (COVID-19) pandemic (Droesch, 2020). While some scholars argue esport is a contemporary sport (Thiel & John, 2018), others reject the idea of competitive gaming as sport (Borggrefe, 2018). Scholars see chances and benefits but also threats and risks for society and the depiction of sport on both sides (Jonasson & Thiborg, 2010; Pfeffel, Horn, Nickolai, & Ratz, 2020; Willimczik, 2019b). This ambiguity can also be seen on a political level since some countries regard esport as a sport, while others have not reacted yet or refuse this acknowledgement (Pack & Hedlund, 2020). Still, the amount of research on esport is growing and topics shift from explanations or translations of esport’s nature, towards more precise ones (Reitman, Anderson-Coto, Wu, Lee, & Steinkuehler, 2020), like the link between nonverbal communication (Leavitt, Keegan, & Clark, 2016) or team composition (Goyal, Sapienza, & Ferrara, 2018), and in-game performance. Scholars have been raising the question on what societal impact esport has, e.g., the influence of and effects on various areas of society and how it is taking root (Holmberg, Bowman, Bowman, Didegah, & Kortelainen, 2019), but this is yet to be investigated (Bascón-Seda & Rodríguez-Sánchez, 2020; Jonasson & Thiborg, 2010). The present study examines this issue in form of a scoping review, since it allows handling a broad research question, identifying the extent of research for a specific topic, summarizing and disseminating findings, mapping out key concepts, and analyzing emerging evidence as a foundation for prospective research (Arksey & O’Malley, 2005; Peters et al., 2017). The aim of this study is to examine the societal impact of esport, consolidate discussions about the topic, offer a deeper base for constructive debates and contribute to research evaluating esport’s impact on society.

## Theoretical background

This section defines the terms esport and societal impact and concludes with the theoretical framework for this study, the Mapping Elite Sport Societal Impact (MESSI) model (De Rycke & De Bosscher, 2019), which is used to investigate societal impact of sport and is considered an adequate approach for studies on the topic (De Bosscher et al., 2021; De Rycke & De Bosscher, 2020; De Rycke, De Bosscher, Funahashi, & Sotiriadou, 2019).

### What is esport?

There appears to be no grammatical consensus about a common terminology of electronic sport. Common expressions are e‑sport, esport, cybersport or pro gaming, an abbreviation for professional gaming and a professional competitor or athlete being called pro gamer. Along with the heterogeneous terminology various definitions have evolved since the first appearance of the term esport in 1999 (Wagner, 2006). There is a characteristic distinction between game-related and sport-related definitions. Game-related definitions highlight a certain degree of organization and competitiveness of digital, online, virtual, computer, or video gaming (Borowy & Jin, 2013; Maric, 2011; Weiss & Schiele, 2013; Witkowski, 2012). Sport-related definitions refer to typical characteristics usually connoted to sportive competitions, like physical and mental prowess being applied in a digital environment or under the use of information and communication technologies (Hemphill, 2005; Wagner, 2006). While none of these definitions are mutually exclusive, they share certain commonalities. Therefore, esport can be described as competitive and organized computer and video gaming, in which two or more parties (individuals or teams) face each other under regulated and balanced conditions. It takes strategical, tactical, physical, and mental skill to outperform the opponent.

### Elaborating the societal impact of sport

Although societal and social impact are often used synonymously, there are differences between the terms (Bornmann, 2013). Social impact refers to positive effects being triggered directly or indirectly on a personal level by an intervention or an entity. Societal impact includes all effects on several areas of society, understanding how an entity is rooted in society, with both positive, therefore including social impact, and negative consequences (Holmberg et al., 2019; Vanclay, Esteves, Aucamp, & Franks, 2015). Therefore, this review focuses on the latter.

The societal impact of sport is a ubiquitous topic in sport science. The positive effects of sport in form of physical activity for mental and physical health are well documented (Eime, Young, Harvey, Charity, & Payne, 2013; Warburton & Bredin, 2016). Beyond physical activity, research on societal impact of sport offers insights into handling decisions about sport interventions, such as funding, hosting events, health care, socialization, economic development, and many more (Lawson, 2005; Pawlowski et al., 2018; Tonts, 2005). This also indicates that the way sport is managed affects society (Chalip, 2006; Taks, Chalip, & Green, 2015): There are strategically desirable impacts on different societal levels when managing sport with positive effects, like increasing of subjective well-being due to hosting sport events, but also negative side effects like financial risk and opportunity costs (Cornelissen & Maennig, 2010; Kavetsos & Szymanski, 2010; Schulenkorf, 2009). To identify sport’s societal impact De Rycke and De Bosscher (2019) conducted a mapping review based on 391 empirical studies and developed the MESSI model. They clustered 128 isolated topics in 79 subcategories and assigned them to 10 superordinate categories, each distinguishing positive and negative impacts (Table 1). Although the model focuses only on elite sport, considering a demonstration effect, elite sport can also affect sport on grass-roots or amateur levels, in terms of participation, engagement or subjective well-being (Kavetsos & Szymanski, 2010; Weed et al., 2015). Therefore, impact beyond the elite sport level can be observable.

**Table 1:** Depiction of the Mapping Elite Sports’ potential Societal Impact (MESSI) framework, showing areas in which societal impact of sport can be observed, modified by adding numberings to categories and subcategories (De Rycke & De Bosscher, 2019)

Context	Events/Athletes & Teams/Successes/Stakeholders worldcups, Olympic games, championships/athletes, football players, sports teams/winning medals, games, records/coaches, sport organisations, sponsors									
Category	(1) Social equality & inclusion	(2) Collective identity & pride	(3) Ethics & fair play	(4) Feel good & passion	(5) Fans & media attraction	(6) Prestige & image	(7) Athletes ability & quality of life	(8) Sport participation & health	(9) Sponsors & commercial activity	(10) Local consumption & living conditions
Potential positive impacts	(1) Integration (2) Social Equality (3) Inclusion (4) Social Justice (5) Socioeconomic equality	(1) Community’ identity (2) Community’ pride (3) Socializing opportunities	(1) Ethics (2) Symbolism & Rituals (3) Fair play (4) Social debate	(1) Pleasure (2) Special experiences (3) Well-being (4) Passion	(1) Beauty’ of sport (2) Fandom (3) Celebrities (4) Media Consumption (5) Sport Knowledge	(1) Globalization (2) International Prestige (3) Political Power (4) Peace building (5) Country/city marketing	(1) Fame (2) Role model function (3) Quality of life (4) Life skills	(1) Identification (2) Sport participation (3) Volunteering (4) Adoption qualities (5) Health awareness	(1) Economic boost (2) Sponsorship (3) Media rights (4) Sport industry assets (5) Commercial activity (6) Innovation (7) Fundraising	(1) Consumption (2) Employment (3) Tourism (4) (sport) infrastructure (5) Greening
Potential negative impacts	(6) Sexism (7) Exclusion (8) Exploitation (9) Discrimination	(4) Opposition *&* rivalry (5) Chauvinism (6) Shame	(5) Corruption & Fraud (6) Hooliganism (7) Deviant examples	(5) Disappointment (6) Failure	(6) Gambling (addiction) (7) Repulsion (8) Drop sports’ image	(6) Soft power (7) Bad international image (8) War propaganda	(5) Pressure (6) Injuries (7) Safe guarding issues (8) Post-career depression (9) Doping	(6) Discouragement effect (7) Unhealthy lifestyle (8) Distorted body image	(8) Associations with scandals (9) Financial hangover	(6) Legacy costs (7) Environmental impact (8) Declined living conditions (9) Excessive Investments

## Methodology

The present review follows the preferred reporting items for systematic reviews and meta-analysis extension for scoping reviews (PRISMA-ScR) guidelines (Tricco et al., 2018). It optimizes the methodological precision, rigor, and quality compared to the classic approach of scoping reviews introduced by Arksey and O’Malley (2005). Objectives, inclusion criteria, and method of this review were specified and documented in a protocol in advance (https://osf.io/s98fc). Any divergence from the protocol is noted in the following section.

### Search strategy

In this scoping review MESSI serves the purpose of identifying key terms which come to use in the search strategy to match the topic of esport with a model for the evaluation of societal impact (Table 2). Conducting the search, set (1) is matched with the categories of societal impact (2) to (11) and their respective subcategories. Thus, it is possible to identify publications which identify traits of esport regarding the corresponding fields and eventually allow insights on the potential societal impact. Some of the concepts or terms used as the (sub-)categories, appear to be somewhat too abstract in the work of De Rycke and De Bosscher (2019) for using them as search terms. Therefore, scholars recommend to adjust said terminology and use search operators to increase methodological rigor (Kugley et al., 2016). Overall, this results in a heterogeneous search strategy which fits the scoping review approach, for it does not call for a deep dive into the topic but examine it on a broad level. For the same reason no publication date limitation is set. The search was conducted on 13 December 2020. Arksey and O’Malley (2005) propose four steps for conducting the search, which are slightly altered for this study: (1) Searching electronic journal databases EBSCOHost, PubMed, Web of Science, and SagePub; (2) searching in reference lists of eligible studies; (3) additional research with GoogleScholar and hand-searching of key journals to ensure no paper is omitted and find further insights into grey literature; (4) searching in existing networks, relevant organizations, and conferences.

**Table 2:** Search terms for literature search adjusted to fit the EBSCOHost database

Set	Search Terms
#1	Electronic sport* OR ″e-sport*″ OR ″esport*″ OR ″cybersport″ OR ″professional gam*″ OR ″pro gam*″ OR ″competitive gam*″
#2	Integration OR ″social equality″ OR ″equality″ OR ″socio-economic equality″ OR ″justice″ OR ″social justice″ OR ″inclusion″ OR ″sexism″ OR ″exclusion″ OR ″exploitation″ OR ″discrimination″
#3	″community identity″ OR ″collective identity″ OR ″identity″ OR ″community pride″ OR ″pride″ OR ″social* opportunit*″ OR ″opposition″ OR ″rival*″ OR ″chauvin*″ OR ″shame*″
#4	″ethic*″ OR ″symbo*″ OR ″ritual*″ OR ″fair play″ OR ″sportsmanship″ OR ″social debate″ OR ″corrupt*″ OR ″fraud″ OR ″hooligan*″ OR ″deviant example*″ OR ″devian*″
#5	Pleasure OR ″special experience*″ OR ″well-being″ OR ″feel good″ OR ″passion″ OR ″disappoint*″ OR ″fail*″
#6	Beauty N5 ?sport OR ″media attraction″ OR ″fandom″ OR ″fan″ OR ″celebrit*″ OR ″media consum*″ OR ″?sport knowledge″ OR ″gam* addict*″ OR ″addict*″ OR ″repuls*″
#7	Globali?ation OR ″prestige″ OR ″polit* power″ OR ″peace* build*″ OR ″marketing″ OR ″soft* power*″ OR ″image″ OR ″propaganda″
#8	Athletes ability OR ″fame″ OR ″role model″ OR ″quality N5 life″ OR ″life skill*″ OR ″pressure″ OR ″injur*″ OR ″safeguarding″ OR ″depressi*″ OR ″doping″ OR ″cheat*″
#9	Identification OR ″participation″ OR ″volunteering″ OR ″adoption qualit*″ OR ″health awareness″ OR ″health″ OR ″discouragement effect″ OR ″unhealthy lifestyle″ OR ″body image″
#10	Economic boost OR ″sponsor*″ OR ″media right*″ OR ″?sport industry″ OR ″commerc*″ OR ″innovation″ OR ″fundrais*″ OR ″scandal*″ OR ″financial risk″
#11	Consum* OR ″employ*″ OR ″touris*″ OR ″infrastructure″ OR ″greening″ OR ″legacy cost*″ OR ″environment*″ OR ″living condition*″ OR ″invest*″
#12	“(1) AND ((2) OR (3) OR (4) OR (5) OR (6) OR (7) OR (8) OR (9) OR (10) OR (11))”

### Selection process and data extraction

**Fig. 1:**
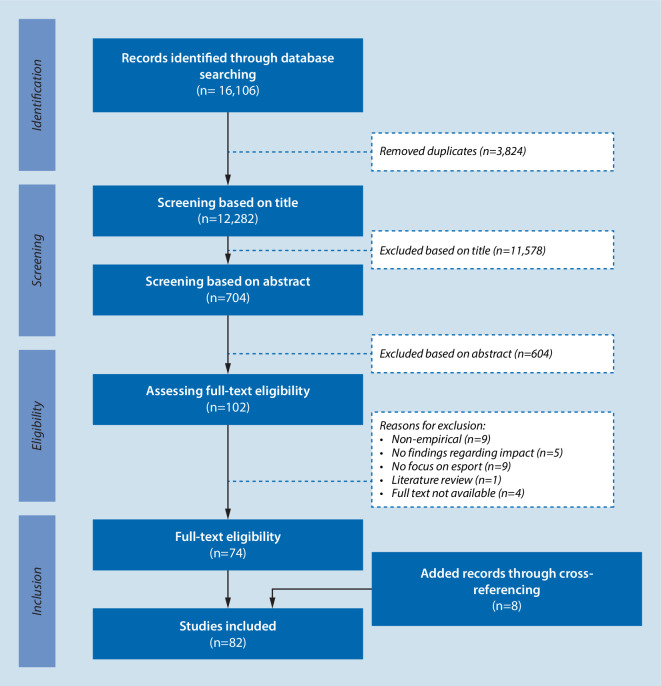
Flow diagram adapted from the PRISMA-ScR guidelines (Tricco et al., 2018)

Literature fitting the following criteria are eligible for the study: (1) qualitative, quantitative, and mixed-method research studies (both observational and experimental); (2) conference and workshop proceedings; (3) theses; (4) unpublished work; (5) grey literature; (6) published in English, French, German, Spanish, and Italian; (7) full-text availability. Studies are excluded if they were non-empirical (reviews, editorials, comments, essays, etc.), they do not discuss esport according to the study’s definition, or the search terms are not discussed as intended within the framework. Articles are first scanned by title, then by abstract, and lastly by full text Fig. 1. If an article does not meet the inclusion criteria it is not further taken note of. It is recommended to sift the articles with at least two reviewers to increase methodical rigor (Tricco et al., 2018; von Elm, Schreiber, & Haupt, 2019). Any disagreement is settled via constructive debating. The data extraction tool described in the protocol has been modified throughout the process. Extracted data included author, year, origin, aim, study design, sample characteristics and assignment to the review’s framework.

## Findings

The initial search yielded a total of 16,106 articles, with 12,282 remaining after removing duplicates. After screening and cross-referencing 82 articles were eligible.

Although the earliest study included was publicized in 2005, most publications were published in recent years. More than half of all publications are from the years 2019 (*n* = 15, 18.52%) and 2020 (*n* = 34, 41.98%). While there are studies included from 26 nations, more than half are from USA (*n* = 28, 34.57%), Spain (*n* = 10, 12.35%) and Finland (*n* = 8, 9.88%), while 10.98% are of far eastern origin (*n* = 9). A total of 17 different methods are used in the studies, with the majority of 71.95% (*n* = 59) applying one methodology and 28.05% (*n* = 23) studies applied a mixed-method approach including two or three different data assessment tools. Most studies gathered data using quantitative surveys (*n* = 51, 62.96%) or qualitative interviews (*n* = 23, 28.40%). Four studies used quantitative surveys and qualitative interviews, three used observations and quantitative surveys, and two applied observations and qualitative interviews. Furthermore, quantitative surveys were combined once with MRI (magnetic resonance imaging) scans, once with exploratory data analysis and twice with exploratory field research. Qualitative interviews were applied twice with focus groups and once with a netnographic analysis. There are eight studies each using three tools, of which two applied quantitative surveys, qualitative and observations, two used observations, qualitative interviews, and document analysis, two combined MRI scans with qualitative interviews and a quantitative survey and one combined two types of document analysis with qualitative interviews. Sample sizes from studies including quantitative surveys ranged from 23 (Hyun et al., 2013) to 68,539 (Karakus, 2015). Qualitative interview studies included four (Bertschy, Mühlbacher, & Desbordes, 2020; Mühlbacher & Bertschy, 2020) to 35 test persons (Lin & Zhao, 2020; Zhao & Lin, 2020). The age of the sample sizes ranged from eight (Lobel, Engels, Stone, & Granic, 2019) to 80 (Macey, Abarbanel, & Hamari, 2020). Only six studies included more female probands than males. All publications can be assigned to the ten categories of the framework and their respective subcategories. More than half of them (*n* = 43, 52.44%) address one category. The remaining 39 studies can be matched with two to five categories each. Regarding the subcategories nearly a third cover one topic (*n* = 27, 32.93%) and the majority covering two (*n* = 30, 36.59%). In sum, 47 different subcategories of all ten categories are treated. One study can be assigned to four categories treating seven subcategories (Seo, 2016), another treats six subcategories under five categories (Schaeperkoetter et al., 2017). Most studies can be assigned to the categories Sport Participation & Health (29 times), Collective Identity & Pride (20 times) and Fans & Media Attraction, while Prestige & Image (8 times), Sponsors & Commercial Activity (8 times) and Local Consumption & Living Conditions (3 times) are the least covered topics. The most frequent subcategories treated are sport participation in 19 studies, socializing opportunities in 18 studies, media consumption in 12 studies and health awareness in ten. Because of the variety of topics addressed, it is difficult to depict in depth findings across all studies. Therefore, findings for each category are treated in the following subsections. As most studies cover more than one subcategory, they are reported multiple times in the next section. Table 3 summarizes all studies and which topics are treated across all studies.

**Table 3:** Categories and subcategories covered across all studies

Category (*n*; %)	Subcategory	*n* (%)	Study
Social Equality & Inclusion (*n* = 13; 15.85%)	Integration	1 (1.22%)	Freeman & Wohn, 2017
	Social Equality	1 (1.22%)	Taylor & Stout, 2020
	Inclusion	4 (4.88%)	Hayday & Collison, 2020; McCauley, Tierney, & Tokbaeva, 2020; Pizzo, Jones, & Funk, 2019; Xue, Newman, & Du, 2019
	Sexism	5 (6.10%)	Hayday & Collison, 2020; Ratan, Taylor, Hogan, Kennedy, & Williams, 2015; Ruvalcaba, Shulze, Kim, Berzenski, & Otten, 2018; Taylor & Stout, 2020; Xue et al., 2019
	Exclusion	6 (7.32%)	Hayday & Collison, 2020; Jansz & Martens, 2005; Ruvalcaba et al., 2018; Schaeperkoetter et al., 2017; Taylor & Stout, 2020; Xue et al., 2019
	Discrimination	4 (4.88%)	Hayday & Collison, 2020; Kwak, Blackburn, & Han, 2015; Mattinen & Macey, 2015; Peng, Dickson, Scelles, Grix, & Brannagan, 2020
Collective Identity & Pride (*n* = 24; 29.27%)	Community identity	8 (9.76%)	Fiskaali, Lieberoth, & Spindler, 2020; Freeman & Wohn, 2017; Jang, Kim, & Byon, 2020; Jansz & Martens, 2005; Pizzo et al., 2019; Seo, 2016; Whalen, 2013; Xue et al., 2019
	Socializing opportunities	18 (21.95%)	Baltezarević & Baltezarević, 2019; Fiskaali et al., 2020; Freeman & Wohn, 2017; Jansz & Martens, 2005; Karsenti & Bugmann, 2018; Lee, Lin, Teo, Tan, Lin, & Acm., 2018; Lobel et al., 2019; McCauley et al., 2020; Pizzo et al., 2018; Qian, Wang, Zhang, & Lu, 2020b; Schaeperkoetter et al., 2017; Seo, 2016; Trepte, Reinecke, & Juechems, 2012; Weiss, 2011; Weiss & Schiele, 2013; Whalen, 2013; Wohn & Freeman, 2020; Xiao, 2020
	Opposition & rivalry	1 (1.22%)	Hayday & Collison, 2020
	Chauvinism	3 (3.66%)	Hamari & Sjöblom, 2017; Ratan et al., 2015; Xue et al., 2019
Ethics & fair play (*n* = 11; 13.41%)	Ethics	1 (1.22%)	Seo, 2016
	Symbolism & Rituals	1 (1.22%)	Schaeperkoetter et al., 2017
	Fair play	5 (6.10%)	Baltezarević & Baltezarević, 2019; Brown, Billings, Murphy, & Puesan, 2018; Martončik, 2015; Seo, 2016; Whalen, 2013
	Social debate	1 (1.22%)	Tjønndal, 2020
	Deviant examples	4 (4.88%)	Adachi & Willoughby, 2011; Adachi & Willoughby, 2013; Choi, Hums, & Bum, 2018; Schmierbach, 2010
Feel good & passion (*n* = 11; 13.41%)	Pleasure	2 (2.44%)	Jang et al., 2020; Seo, 2016
	Special experiences	2 (2.44%)	Jang et al., 2020; Martončik, 2015
	Well-being	2 (2.44%)	Baltezarević & Baltezarević, 2019; Fiskaali et al., 2020
	Passion	8 (9.76%)	Bertran & Chamarro, 2016; Choi, 2019; Garcia-Lanzo & Chamarro, 2018; Jang et al., 2020; Lee et al., 2018; Macey & Hamari, 2018; Pizzo et al., 2018; Seo, 2016
Fans & media attraction (*n* = 20; 24.39%)	Fandom	5 (6.10%)	Brown et al., 2018; Choi, 2019; Karakus, 2015; Kim & Kim, 2020; Xiao, 2020
	Celebrities	1 (1.22%)	Ward & Harmon, 2019
	Media consumption	12 (14.63%)	Brown et al., 2018; Choi, 2019; Hamari & Sjöblom, 2017; Kim & Kim, 2020; Lee & Schoenstedt, 2011; Macey et al., 2020; Mangeloja, 2019; Qian, Wang, & Zhang, 2020a; Qian et al., 2020b; Qian, Zhang, Wang, & Hulland, 2020c; Wohn & Freeman, 2020; Xiao, 2020
	Sport knowledge	1 (1.22%)	Brown et al., 2018
	Gambling (addiction)	6 (7.32%)	Bertran & Chamarro, 2016; Choi et al., 2018; Macey et al., 2020; Macey & Hamari, 2019; Sweeney, Tuttle, & Berg, 2019; Whalen, 2013
	Drop sports’ image	2 (2.44%)	Hou, Yang, & Panek, 2020; Macey et al., 2020
Prestige & Image (*n* = 8; 9.76%)	Globalization	5 (6.10%)	García & Murillo, 2020; Parshakov, Paklina, Coates, & Chadov, 2020; Postigo Fuentes & Fernández Navas, 2020b; Postigo Fuentes & Fernández Navas, 2020a; Ward & Harmon, 2019
	International Prestige	2 (2.44%)	Lin & Zhao, 2020; Pizzo et al., 2019
	Political Power	1 (1.22%)	Lin & Zhao, 2020
	Country/city marketing	2 (2.44%)	Lin & Zhao, 2020; Zhao & Lin, 2020
	Soft power	2 (2.44%)	Lin & Zhao, 2020; Pizzo et al., 2019
	War propaganda	1 (1.22%)	Lin & Zhao, 2020
Athletes ability & quality of life (*n* = 12; *n* = 14.63%)	Fame	1 (1.22%)	Ward & Harmon, 2019
	Role model function	3 (3.66%)	Kari & Karhulahti, 2016; Schaeperkoetter et al., 2017; Kari, Siutila, & Karhulahti, 2019
	Life skills	8 (9.76%)	Baltezarević & Baltezarević, 2019; Freeman & Wohn, 2017; Lobel et al., 2019; Nielsen & Hanghoj, 2019; Paravizo & de Souza, 2019; Postigo Fuentes & Fernández Navas, 2020b; Postigo Fuentes & Fernández Navas, 2020a; Seo, 2016
	Pressure	2 (2.44%)	Paravizo & de Souza, 2019; Perez-Rubio, Gonzalez, & Garces de los Fayos, 2017
	Post-career depression	1 (1.22%)	Perez-Rubio et al., 2017
Sport participation & health (*n* = 29; 35.37%)	Identification	3 (3.66%)	Karsenti & Bugmann, 2018; Pizzo et al., 2019; Schaeperkoetter et al., 2017
	Sport participation	19 (23.17%)	Abbasi, Nisar, Rehman, & Ting, 2020; Adachi & Willoughby, 2011; Adachi & Willoughby, 2013; García & Murillo, 2020; Gray, Vuong, Zava, & McHale, 2018; Jang & Byon, 2019; Jang & Byon, 2020; Jansz & Martens, 2005; Kwak, Hwang, Kim, & Han, 2020; Lobel et al., 2019; Marcano Lárez, 2012; Matuszewski, Dobrowolski, & Zawadzki, 2020; Rudolf et al., 2020; Schaeperkoetter et al., 2017; Schmierbach, 2010; Stankovic & Kostadinovic, 2017; Trotter, Coulter, Davis, Poulus, & Polman, 2020; Weiss, 2011; Weiss & Schiele, 2013
	Adoption qualities	5 (6.10%)	Hagiwara, Akiyama, & Takeshita, 2019; Hyun et al., 2013; Kari et al., 2019; Karsenti & Bugmann, 2018; Matuszewski et al., 2020
	Health awareness	10 (12.20%)	Bayraktar, Yıldız, & Bayrakdar, 2020; DiFrancisco-Donoghue, Balentine, Schmidt, & Zwibel, 2019; DiFrancisco-Donoghue, Werner, Douris, & Zwibel, 2020; Gray et al., 2018; Hagiwara et al., 2019; Hyun et al., 2013; Kari & Karhulahti, 2016; Kwak et al., 2020; Peng et al., 2020; Trotter et al., 2020
	Unhealthy lifestyle	3 (3.66%)	DiFrancisco-Donoghue et al., 2019; DiFrancisco-Donoghue et al., 2020; Kwak et al., 2020
Sponsors & commercial activity (*n* = 8; 9.76%)	Economic boost	1 (1.22%)	Zhao & Lin, 2020
	Sponsorship	2 (2.44%)	Abreu Freitas, Contreras-Espinosa, & Correia, 2020; Elasri-Ejjaberi, Rodriguez-Rodriguez, & Aparicio-Chueca, 2020
	Commercial activity	7 (8.54%)	Bertschy et al., 2020; Elasri-Ejjaberi et al., 2020; Karakus, 2015; Mühlbacher & Bertschy, 2020; Peng et al., 2020; Wohn & Freeman, 2020; Zhao & Lin, 2020
Local consumption & living conditions (*n* = 3; 3.66%)	Consumption	1 (1.22%)	Jang et al., 2020
	Tourism	2 (2.44%)	McCauley et al., 2020; Vegara-Ferri, Ibáñez-Ortega, Carboneros, López-Gullón, & Angosto, 2020
	Sport infrastructure	1 (1.22%)	McCauley et al., 2020

### (1) Social equality and inclusion.

A total of 13 studies covered topics related to the first category. Studies delivered insights on integration (Freeman & Wohn, 2017), promoting social equality (Taylor & Stout, 2020), and inclusion (Hayday & Collison, 2020; McCauley et al., 2020; Pizzo et al., 2019; Xue et al., 2019), as it is a platform for like-minded people regardless of their origin, gender or (dis-)abilities. One study reveals how normative gender-roles exist in esport and can therefore lead to the opposite of the aforementioned, despite theoretical accessibility and equal opportunities, as there is no skill difference between males and females in esport (Ratan et al., 2015). Several studies thematize condescending behavior towards women like sexist behavior and exclusion, namely harassment or male hedonism (Jansz & Martens, 2005; Ratan et al., 2015; Ruvalcaba et al., 2018), low acceptance of other genders and lacking political correctness (Hayday & Collison, 2020; Xue et al., 2019), and application of gender normative roles in games (Ratan et al., 2015). On collegiate or amateur level, there are barely programs or approaches which tackle discriminatory and exclusive issues (Taylor & Stout, 2020). Generally, discriminatory behavior is becoming an overarching problem for sports that are consumed mainly online and anonymously, which is even intensified in casual gaming and semi-professional esport due to its anonymous exertion (Hayday & Collison, 2020; Kwak et al., 2015; Mattinen & Macey, 2015; Peng et al., 2020). Players, willing to go pro, who do not see their performance being recognized also sense a feeling of exclusion (Schaeperkoetter et al., 2017).

### (2) Collective identity and pride.

Community identity is evolving and growing among esport enthusiasts (Fiskaali et al., 2020; Freeman & Wohn, 2017; Pizzo et al., 2019; Seo, 2016; Xue et al., 2019). This can be fostered by attending live events or LAN-parties (Jang et al., 2020; Jansz & Martens, 2005; Whalen, 2013), which also contribute to the findings that esport is a platform for socializing opportunities. This can occur in dedicated live events (Jang et al., 2020; Jansz & Martens, 2005; McCauley et al., 2020; Whalen, 2013) or generally by engaging in the esport environment both online and offline (Baltezarević & Baltezarević, 2019; Fiskaali et al., 2020; Freeman & Wohn, 2017; Karsenti & Bugmann, 2018; Lee et al., 2018; Lobel et al., 2019; Pizzo et al., 2018; Qian et al., 2020b; Schaeperkoetter et al., 2017; Seo, 2016; Trepte et al., 2012; Weiss, 2011; Weiss & Schiele, 2013; Wohn & Freeman, 2020; Xiao, 2020). However, esport enthusiasts try to distinguish themselves based on the game they play (Karakus, 2015; Kim & Kim, 2020), which results in tribal behavior among the different player bases (Hayday & Collison, 2020). Furthermore, various chauvinistic tendencies in esport can be observed regarding gender (Hamari & Sjöblom, 2017; Ratan et al., 2015; Xue et al., 2019).

### (3) Ethics and fair play.

Specific ethics, norms, and codices such as fair play, sportsmanship, and respect for the opponent are crucial elements of esport (Baltezarević & Baltezarević, 2019; Brown et al., 2018; Martončik, 2015; Seo, 2016), although they can occur in distinguished manner compared to traditional sport (Whalen, 2013). This also expresses itself through the fact that esport players see themselves as athletes (Schaeperkoetter et al., 2017). The comparison with traditional sport however sparks debates about potential threats coming from esport towards traditional sport and society because it undermines the physical connotation and threatens its worthiness of financial support (Tjønndal, 2020). Four studies investigate the relationship between competitive video or computer games and aggressive behavior, concluding that competition, not violent or explicit content, leads to aggressive behavior (Adachi & Willoughby, 2011, 2013; Choi et al., 2018; Schmierbach, 2010).

### (4) Feel good and passion.

Engaging in esport, both passive and active consumption, is seen as pleasureful and special experience by enthusiasts (Jang et al., 2020; Martončik, 2015; Seo, 2016), raising well-being among peers (Baltezarević & Baltezarević, 2019; Fiskaali et al., 2020). Esport players show both obsessive (Macey & Hamari, 2018) and harmonious passion (Garcia-Lanzo & Chamarro, 2018; Jang et al., 2020; Lee et al., 2018; Pizzo et al., 2018; Seo, 2016) with the former predicting problematic gaming behavior and the latter being a protection from negative consequences (Bertran & Chamarro, 2016; Choi, 2019).

### (5) Fans and (media) attraction.

Like in traditional sports, fandom expresses itself by loyalty towards players and teams (Brown et al., 2018; Choi, 2019; Xiao, 2020), but fans also feel a strong loyalty, towards their favorite esport title (Hayday & Collison, 2020; Karakus, 2015; Kim & Kim, 2020). Ward and Harmon (2019) identify superstar economics establishing in esport, like in traditional sport, music, or acting. Twelve studies deliver insights on media consumption in esport, several of which show that esport consumption motives are similar to traditional sport consumption, like socialization, fandom and acquiring game related knowledge (Brown et al., 2018), fandom and uncertainty of outcome (Mangeloja, 2019), drama, escapism, and aesthetics (Xiao, 2020), competition and peer-pressure (Lee & Schoenstedt, 2011). Choi (2019) distinguishes between fans, passionates and addicts, and shows the different motives for each. Although drama and entertainment are drivers for each type of attachment, escapism is a motive for addicts. Qian et al. (2020b) highlight a slight divergence of esport consumption motives and name skill improvement, appreciation, vicarious sensation, and socializing opportunities as main motives. Hamari and Sjöblom (2017) describe escapism, acquiring knowledge, novelty—such as new teams and players emerging—and enjoyment of aggressive behavior as motives. There are motives that initially developed through esport and gaming context which go beyond traditional sport consumption motivation, like chat rooms included in the stream, personality traits of the streamer, virtual rewards, or the quality of streams (Qian et al., 2020a, c). Streamers as a distinctive feature of esport consumption is also mentioned by Wohn and Freeman (2020). Furthermore, Xiao (2020) observes that spectators tend to watch esport alone, rather than in company. Two studies show that spectators experience flow and subjective well-being (Kim & Kim, 2020) or a sense of achievement (Choi, 2019), during and after the consumption of esport events. Esport consumption can also be an indicator for gambling and eventually gambling disorder, mainly for young males (Macey et al., 2020; Macey & Hamari, 2019). The esport gambling and betting market is currently barely arbitrated, therefore, susceptible to irregularities, match fixing, or betting abuse (Sweeney et al., 2019). Addictive gaming behavior in the context of esport is treated thrice, indicating that a risk of developing gaming disorder or addiction, heavily depends on psychological and social factors of the consumer, not necessarily by the games themselves (Bertran & Chamarro, 2016; Choi et al., 2018; Whalen, 2013). Overall, media attraction of esport is rising and differentiating throughout the past two decades with more positive coverage on the topic (Hou et al., 2020), and scholars argue that esport is becoming mainstream (Macey et al., 2020).

### (6) Prestige and image.

Although esport is a global phenomenon, contributing to international communication in competition (Postigo Fuentes & Fernández Navas, 2020a, b), especially for the younger male generations (García & Murillo, 2020), there is a divergence in popularity of esport (Parshakov et al., 2020) and genres or games played as esport (Hayday & Collison, 2020; Karakus, 2015; Kim & Kim, 2020; Ward & Harmon, 2019) in different nations and regions worldwide. Two studies find that esport players and teams can be used to obtain prestige for a certain cause whether it be representing a university (Pizzo et al., 2019) or a nation (Lin & Zhao, 2020). Furthermore, studies imply that esport is used to propagate political power or create nationalism based on a meritocratic neoliberalist approach where whoever outperforms his opponents earns the right to represent and bring glory to the home country (Lin & Zhao, 2020; Zhao & Lin, 2020).

### (7) Athletes’ ability and quality of life.

Regarding the characterization of esport athletes, studies show how up and coming esport players thrive to become professionals and identify as athletes (Schaeperkoetter et al., 2017). Ward and Harmon (2019) indicate that “superstardom” exists in esport and esport players can act as role models. Eight studies conclude that playing games competitively helps to improve communicative skills (Nielsen & Hanghoj, 2019; Paravizo & de Souza, 2019), social interaction among peers and problem solving skills (Baltezarević & Baltezarević, 2019; Lobel et al., 2019), and soft skills (Freeman & Wohn, 2017). Esport, due to the internationality, helps to improve foreign language skills (Postigo Fuentes & Fernández Navas, 2020a, b). On the other hand, one study shows that pro gamers endure pressure from their team or organization, the fans, and themselves (Paravizo & de Souza, 2019), while another describes the danger of burn-out on a professional level (Perez-Rubio et al., 2017).

### (8) Sport participation and health.

Most findings can be matched to this category, with its subcategories being treated 40 times in total by 29 studies. People who play esport on an organized competitive level identify as athletes (Karsenti & Bugmann, 2018; Pizzo et al., 2019; Schaeperkoetter et al., 2017). From a demographic perspective, esport is predominantly played by young males (García & Murillo, 2020; Jansz & Martens, 2005; Lobel et al., 2019; Marcano Lárez, 2012; Rudolf et al., 2020; Stankovic & Kostadinovic, 2017). Competitive gaming can lead to short-term aggressive behavior, regardless of the game played and whether it contains violence (Adachi & Willoughby, 2011, 2013; Schmierbach, 2010), short-term boost of concentration (Hagiwara et al., 2019) and after a certain duration increase testosterone, dehydroepiandrosterone and androstenedione (Gray et al., 2018). Furthermore, it fulfills hedonistic needs like escapism or competitive needs like challenge or competition (Jang & Byon, 2019, 2020; Weiss, 2011; Weiss & Schiele, 2013). Quantitative survey studies show increased social capital among esport players (Schaeperkoetter et al., 2017) and improved behavioral and emotional status (Kwak et al., 2020). A mixed method study finds improved team behavior knowledge among esport players (Karsenti & Bugmann, 2018). Players of higher level are more determined, less agreeable and less extroverted than low level players (Matuszewski et al., 2020); however, agreeableness and extroversion as well as consciousness and openness to experience are described as triggers of esport consumer engagement (Abbasi et al., 2020). Higher time spent playing, positively correlates with performance level and with physical activity (Trotter et al., 2020) and career length of professional StarCraft gamers correlates with cortical thickness in three brain regions, with the frontal gyrus positively correlating with rate of winning (Hyun et al., 2013). Two studies show that pro gamers perform above average physical exercise than recommended by the World Health Organization, as they consider it to help their competitive strength (Kari & Karhulahti, 2016; Kari et al., 2019). Contrary to these findings, two studies find a connection between esport activity and reduced physical activity with negative effects regarding the players’ body composition (Bayraktar et al., 2020; DiFrancisco-Donoghue et al., 2020). DiFrancisco-Donoghue et al. (2019) conclude that esport sees similar clinical pictures as sedentary desk jobs. Another study shows how the esport community can oppose a threat to the players mental health, due to toxic behavior and almost no regulatory systems preventing such (Peng et al., 2020).

### (9) Sponsors and commercial activity.

Qualitative and quantitative data show that brands, both sponsors and clubs, enter esport to reach a new younger, mostly male, target group (Bertschy et al., 2020; Elasri-Ejjaberi et al., 2020; Mühlbacher & Bertschy, 2020) and bring added value to consumers and fan experience (Abreu Freitas et al., 2020). Two qualitative studies observe that game developers and publishers are the dominant player in esport, since it is a major revenue business, and it can be used as a marketing tool for games distribution (Peng et al., 2020; Zhao & Lin, 2020). Quantitative data shows how, along with esport, streaming is developing into an essential economic field, which esport players use to earn money from fans by donations or sponsors (Karakus, 2015; Wohn & Freeman, 2020).

### (10) Local consumption and living conditions.

Only three studies provide insights on the tenth category. LAN parties and esport tournaments are popular events among esport-enthusiasts and can enhance touristic value of the host cities (Jang et al., 2020; McCauley et al., 2020; Vegara-Ferri et al., 2020).

## Discussion

This scoping review examines the current state of research regarding literature of esport on the societal impact of esport. It helps to map out the research environment, illustrate key findings, and explore gaps of knowledge. In the past few years, the frequency of studies treating the subject is rising and their origin and the topics are diversifying. This indicates that esport is a popular but still emerging area and field of research. The MESSI framework delivers an adequate approach to contextualize findings into ten categories depicting its potential societal impact. Regarding the categories, the impact of esport seems like the one from traditional sport, although not all potential areas of impact could be addressed. A reason could be that not all categories are transferable to esport (greening, hooliganism) or topics are yet to be studied (corruption, fraud, doping). Eventually, this section derives four areas of key findings, proposes theoretical implication, and states research desiderata.

### Active and passive esport consumption

Esport is consumed for mainly the same reasons as traditional sport but there are a variety of distinctive motives which cannot be found in traditional sport, like participation in chats, or earning virtual goods. Fandom towards players and teams exist as well but loyalty towards the game is also a crucial part of esport fandom. Furthermore, esport players see themselves as athletes and pro gamers show similar traits like professional athletes, in terms of ethics, superstardom, willingness to go pro, or the wish to represent their country as athlete. On the other hand, pro gamers also suffer from fear of failure and pressure from their professional environment. Despite the online nature of esport, local events like tournaments or LAN parties are important occasions for enthusiasts.

### Potential beneficial traits

Despite the negative image and skepticism (Borggrefe, 2018; Pack & Hedlund, 2020; Parry, 2019; Willimczik, 2019a, b), this study’s findings show that esport is a new platform with strong socializing potential for long-established enthusiasts, but also for children and adolescents who see themselves struggling in the conventional sporting world. Although the world of esport has its own values, norms, and behaviors, which beginners are facing, there are no entry barriers for playing and engaging in esport regarding age, gender, sexuality, origin, healthiness, etc. These findings go along with existing propositions of the potential of esport (Heere, 2018). Educational and pedagogical benefits of sport, beyond physical and gross motoric benefits, can be transported, for example to people who are not able to compete in traditional sport. With sport clubs or schools as multiplicators, esports can not only promote communicative skills, fine motoric or cognitive benefits (Jonasson & Thiborg, 2010; Thiel & John, 2018), but also teach media competences and a responsible approach for behavior in digital environments, which is becoming more and more relevant in today’s digitalized society (Thiel & Gropper, 2017).

### Mental and physical health-related issues

The review also shows that behavior among players is barely regulated and can therefore be abused as a platform for verbal discrimination or cyberbullying, which can be harmful to mental health and psychosocial status (Kwan et al., 2020). On a clinical level active esport participation in the population can lead to an increase of esport specific illnesses. On a physical level, intensive playing can lead to lacking physical activity with respective consequences; however, this is not exclusive to esport, but rather to gaming in general (Marker, Gnambs, & Appel, 2019; Schmidt, Kowal, & Woll, 2018). Although the included studies show no clear evidence that esport triggers addictive gaming disorders, the mixed results indicate the relevance of this topic when approaching esport. Eventually, not only in-game mechanics such as virtual item gambling, but also an unregulated esport betting market poses a threat for the young consumer base.

### Popularity of esport

The review shows that both, playing and watching esport is especially popular among young males. Active and passive consumption seem to have a big overlap (Breuer, 2011; McCauley et al., 2020), which can be led back to the complexity of the games or the relatively young existence of esport. However, the popularity of esport differs from nation to nation (Parshakov et al., 2020; Parshakov & Zavertiaeva, 2018). Potential impact needs always to be considered regarding the respective nation and title which is involved in the game. Although there is reason to believe that currently esport does not appear to challenge traditional sport in its popularity, the findings show that esport is becoming mainstream (Macey et al., 2020) and future generations can possibly grow up as fans of esport instead of other sports (Brown et al., 2018; Tjønndal, 2020).

### Theoretical implications

Based on this review’s findings, several implications can be derived. It is necessary to identify potential threats and benefits resulting from the evolution of esport. The esport market is widely unregulated on a governmental level. Up to this point, publishers and game developers are a dominant stakeholder, holding most intellectual property and rights, thus access to esport, with commercial interest. This indicates a potential infiltration of sport structures and systems by the owners of esport titles, usually profit-oriented corporations. Although there are esport associations, based on the model of traditional sport associations, their impact is limited. Other than in traditional sports, where associations function as rule makers, organizer of competitions, and major stakeholder for the sport (Thiel et al., 2013), esport associations are unable to do so, not least because they rely on the collaboration with the publishers, developers and tournament organizers (Pack & Hedlund, 2020). This underlines that esport does not rely on the existing sportive structures but has already created its own ecosystem, where conventional clubs and associations struggle to fit in if they do not manage to adapt (Breuer, 2012). Still, grass-roots sport can open towards esport for both altruistic and economic reason. In sport clubs, esport divisions can help acquiring new target groups and raise awareness for the threats, potentials, and handling of esport and new media in a safe environment not least this fosters the need for socializing opportunities, both off- and online for esport enthusiasts. This could also be used to address the problem of sport drop-outs (Eime, Harvey, & Charity, 2019), or attracting an audience which otherwise would not be interested in joining a club (De Martelaer, van Hoecke, De Knop, Van Heddegem, & Theeboom, 2002; Schmidt et al., 2018). However, including esport in the common sport environment like clubs or in schools must not be seen as a substitution for sports which focus on physical activity, but rather as a supplemental new facet for a post-modern understanding of sport. Furthermore, the positive aspects like socializing opportunities and integrative elements of esport could be used as a healthy approach towards gaming, opposing threats like obsessive gaming for reasons of escapism with negative social and occupational consequences (Kardefelt-Winther, 2014).

### Potential research desiderata

Due to little evidence, mixed results or knowledge gaps, several research desiderata can be identified: (1) why is esport a male-dominated activity, although there are practically no gender barriers; (2) what is the relation between physical activity and both passive and active esport consumption; (3) is there a causality between esport and addictive gaming behavior; (4) what is the origin of frequent discriminatory and toxic behavior in esport and how is it possible to tackle this problem; (5) what role does deviant behavior like doping or cheating play; (6) what impact does esport have in a (sport)political context? When approaching these exemplarily research strands, scholars should also consider, depending on the research question, investigating esport-titles individually, since popularity and requirements can vary considerably.

## Limitations

Conducting the scoping review, there were some limitations that need to be addressed. First, the broad research question and the variety of individual topics being treated reduce the depth of analysis for each of the addressed topics. The demographics of the sample sizes differed significantly; therefore, it was difficult to compare many of the studies with each other. The research landscape can still be considered novel and not as differentiated as for traditional sports. This also manifests itself in 33 out of 79 subcategories of the framework not being treated in this scoping review. Second, due to the scoping review’s nature, quality appraisal was not conducted for the included studies (Arksey & O’Malley, 2005; Tricco et al., 2018). Third, although studies in five languages (German, English, French, Italian and Spanish) were considered, the search was conducted only in English. Furthermore, just a few papers from the Far East, where esport plays a major role, were included due to language restrictions. Fourth, while the selection process was conducted with two researchers, only one author scanned full texts for eligibility. A higher number of researchers could have increased methodological rigor. Fifth, definitions of esport and societal impact are both abstract and can differ depending on the used sources. Although the definition of societal impact and esport, and the MESSI framework used in this review are considered adequate and reasonable, there might be other frameworks and definitions which can be used to investigate the research question. Sixth, nuanced differences between the degree of professionalization are difficult to elaborate regarding certain topics, since the included studies treat all four (i.e., professional, semi-professional, amateur, and casual) gamers.

## Conclusion

The present scoping review provides an overview on the current research of the societal impact of esport focus and shows under which scope esport is yet to be investigated. It can be stated that esport challenges traditional sport and to a certain extent initiates a change of paradigm in sport, which has been predicted by scholars of various fields of research (Cunningham et al., 2018; Heere, 2018). Although it was stated initially that this paper should not be understood as proclamation in favor or against the concept of considering esport as sport, it does intend to shed light on this discussion, underlining arguments from both sides with further insights to adequately extend the knowledge on esport. The findings show that people engage in esport for motives similar to those in traditional sports, but it offers some peculiarities originating from its digital nature which cannot be found in other sports. Still, players consider themselves and behave like athletes, regarding skill or dedication, but also regarding performance pressure. Playing esport can develop communicative, cognitive, and fine motoric skills, but can also lead to physical and mental health risk. Nevertheless, esport is finding its way into the mainstream and will presumably play a more important role in various areas of society. It opens new possibilities for stakeholders from traditional sport like players, clubs, associations, stakeholders from the gaming branch, like publishers, game developers, but also for third party systems, like educational or pedagogical institutions. However, low evidence or gaps regarding some topics shows that the field of research is still very fragmented, and more research is needed to foster existing evidence and develop new insights into the role and impact of esport in society. Because of the intense evolution of esport throughout the past decades, there is still a lot to be learned about it in terms of threats and benefits of this new global sport-like activity, which also shows in the fragmented body of research as certain topics of the framework only being addressed scarcely or not at all. Future research can pick up on this research, test the existing findings and show how its positive or negative manifestations can be guided accordingly.

## Supplementary Information

Preferred reporting items for systematic reviews and meta-analyses extension for scoping reviews (PRISMA-ScR) checklist.

## Declarations

For this article no studies with human participants or animals were performed by any of the authors. All studies performed were in accordance with the ethical standards indicated in each case.
